# Challenges, Opportunities, and Priorities for Advancing Breast Cancer Control in Zambia: A Consultative Meeting on Breast Cancer Control

**DOI:** 10.1200/JGO.18.00222

**Published:** 2019-03-25

**Authors:** Anna Cabanes, Sharon Kapambwe, Susan Citonje-Msadabwe, Groesbeck P. Parham, Kennedy Lishimpi, Tauane A. Cruz, Surendra Shastri

**Affiliations:** ^1^ Susan G. Komen, Washington, DC; ^2^ Ministry of Health, Lusaka, Zambia; ^3^ Cancer Diseases Hospital, Lusaka, Zambia; ^4^ University of North Carolina, Chapel Hill, NC, and University of Zambia, Lusaka, Zambia; ^5^ Susan G. Komen, Dallas, TX; ^6^ University of Texas, MD Anderson Cancer Center, Houston, TX

## Abstract

In 2016, the Zambian government made cancer control a national priority and released a National Cancer Control Strategic Plan for 2016 to 2021, which focuses on malignancies of the breast, cervix, and prostate, and retinoblastoma. The plan calls for a collective reduction in the cancer burden by 50%. In support of this vision, Susan G. Komen sponsored a consultative meeting in Lusaka, Zambia, in September 2017 to bring together the country’s main breast cancer stakeholders and identify opportunities to improve breast cancer control. The recommendations generated during the discussions are presented. There was general agreement that the first step toward breast cancer mortality reduction should consist of implementation of early detection service platforms focused on women who are symptomatic. Participants also agreed that the management of all components of the national breast cancer control program should be integrated and led by the Ministry of Health. As much as possible, early detection and treatment services presently offered by the Cervical Cancer Prevention Program of Zambia and Cancer Diseases Hospital should be leveraged. Efforts are under way through multiple stakeholders to implement the following recommendations: development of national guidelines for the early diagnosis of breast cancer, training of breast surgeons, implementation of early detection and surgical treatment service platforms at the district-hospital level, and epidemiologic research, including the improvement of electronic recording mechanisms.

## INTRODUCTION

Breast cancer is the most common cause of cancer and the leading cause of cancer death in women worldwide.^1^ In low- and middle-income countries, incidence and mortality rates are increasing, and Zambia is no exception.

A 2014 assessment of breast health services in Zambia obtained through a nationwide survey showed that some breast cancer screening and early-diagnosis services exist at the provincial level, but use is low. The capacity of provincial- and tertiary-level facilities to provide cancer services is severely curtailed by a lack of appropriately trained mid- and high-level health personnel and limited pathology services, and advanced therapies (chemotherapy, radiation, and surgery) are concentrated within Lusaka Province.^2^

This report served as the impetus for the implementation of several projects focused on addressing identified gaps. For example, a collaborative training and technical support project, implemented jointly by local partners and a US-based academic center, resulted in the implementation of two new breast cancer clinics in Lusaka.^3^

Recently, the Zambian government made cancer a national priority and released the National Cancer Control Strategic Plan (NCCSP) 2016 to 2021, which focused on four cancers—breast, cervical, and prostate, and retinoblastoma.^4^ The plan is representative of the response to the country’s increasing cancer incidence and is centered around addressing cancer management deficiencies. The plan strives to engage all stakeholders throughout the health care system, including the Ministry of Health, community members, key players, and communities.

Zambia’s current national health strategic plan emphasizes primary health care to achieve universal health coverage for all health services, including cancer. Zambia has prioritized both the control of communicable and noncommunicable diseases, seeking integration of services and taking a multisectoral approach. A key objective is to increase the number of health care workers, with a target of more than 500 specialists by 2021, which will increase the capacity for screening, health promotion, early diagnosis, and treatment across the country. In addition, the National Health Insurance Act was recently passed, and a national health insurance service is being set up that will cover cancer.

**CONTEXT**

**Key Objective**
The objective of the co-creation workshop was to discuss how the Zambian health care community can support the goals of the National Cancer Control Strategic Plan. Instead of implementing best practices that worked elsewhere, a group of breast cancer stakeholders was guided through a co-creation process to collaboratively identify and develop solutions that are desirable, feasible, and viable in the Zambian context.
**Knowledge Generated**
Because early diagnosis must be linked to access to treatment, there was agreement that it is paramount to develop a strategy to decentralize and coordinate services in a single facility, preferably, the district hospital.
**Relevance**
There was consensus that efforts should focus on the early diagnosis of breast cancer to reduce late-stage presentations as a first step toward lowering breast cancer mortality. Initially, priority should be given to the early diagnosis of breast cancer among women presenting with clinical symptoms.

To support the vision of the Ministry of Health and the consistent implementation of innovative cancer-related activities, Susan G. Komen sponsored a consultative meeting to bring together the main breast cancer stakeholders to identify opportunities to improve breast cancer control in Zambia. Among the key players were members of the Cancer Technical Working Group of the Ministry of Health; health care practitioners (nurses, oncologists, pathologists, surgeons), from both the public and private sectors; multilateral organizations; and patient and advocacy organizations.

The objective of the meeting was to discuss feasible activities and initiatives that the Zambian health care community can undertake to reduce breast cancer mortality within the scope of the plan. The strategy was to convene a core group of stakeholders to create (co-create) a set of approaches to the complex problems associated with breast cancer control in general and the unique challenges of breast cancer control specific to Zambia.

The methodology was based on design thinking, a human-centered design discipline that involves placing people at the center of the design and development process, specifically, the people for whom solutions are being designed, such as patients, families, caregivers, and community leaders.^5^

Design thinking is used to help organizations develop sustainable solutions to complex problems. Instead of bringing in best practices that worked elsewhere, leaders and teams are guided through a discovery process in which they develop a shared understanding of the needs, wants, and limitations of the people involved in the challenge. Through co-creation workshops, stakeholders are empowered to collaboratively identify, prototype, and refine potential solutions that are desirable, feasible, and viable.

The variety of techniques and activities used focus on people’s needs, moving from designing for to designing with customers. This brings innovation and creates more support from key stakeholder groups, resulting in a more effective implementation of the resulting solutions and approaches. We hoped that connecting key stakeholders through the co-creation workshop would also provide the necessary ownership for the process of creating a breast cancer strategy, developing services, and delivery of those services.

The goals of the cocreation workshop were:

- To engage stakeholders in developing a collaborative plan that aligns with Zambia’s NCCSP and contributes to its national goals.
- To share feasible and promising practices on breast cancer control and create a platform for scale-up in Zambia.
- To promote a network of expert advocates, including government, private-sector, and traditional medicine practitioners, to design strategies to address breast cancer control.

This report is limited in scope to invited talks and discussions that took place during the consensus meeting in September 2017 in Lusaka. References are included when appropriate, although it is not intended to be a comprehensive review of breast cancer control efforts in Zambia or Africa.

## CHALLENGES AND OPPORTUNITIES IN BREAST CANCER CONTROL IN ZAMBIA

### Most Patients With Breast Cancer Present at Advanced Stages, and Survival Rates Are Low

In Zambia, breast cancer constitutes the second leading cause of cancer mortality after cervical cancer. GLOBOCAN estimates that the incidence of breast cancer in Zambia is 19.9 cases per 100,000 women, and the mortality rate is 8.5 deaths per 100,000 women.^1^

Most patients with breast cancer are diagnosed at advanced disease stages, resulting in limited treatment options and high mortality rates. Among Zambian participants enrolled in an investigation of the determinants of breast cancer mortality in sub-Saharan Africa—the African Breast Cancer Disparities and Outcomes (ABC-DO) study—60% of women presented with late-stage disease.^6^ According to the authors,^6^ this may be explained by limited breast cancer education and awareness, delay in seeking medical services, limited screening, limited diagnostic and treatment services, and a weak referral and follow-up system.

In addition, data on breast cancer stage and molecular profile of tumors are scarce, outside of the ABC-DO study. Recently, Zambia’s National Cancer Registry completed the transformation from a hospital-based to a population-based registry for Lusaka District, with support from the US National Cancer Institute, Union for International Cancer Control, and African Network of Cancer Registries.

Until this year, the national referral hospital for cancer in Zambia—the Cancer Diseases Hospital (CDH)—lacked robust data collection infrastructure and produced little research to inform policy and practice, leaving relevant questions regarding molecular characteristics, histologic subtypes, and breast cancer outcomes unanswered. CDH’s strategic plan for the next few years includes investing in the development of a cancer care data management platform. In 2018, the cancer center received a pilot grant from Susan G. Komen to establish a prospective clinical database to collect and analyze data on its patients with breast and cervical cancer.

### Awareness Levels Among the Zambian Population Are Low

Recent results from the ABC-DO study demonstrate that delayed diagnosis is associated with several modifiable factors, including limited formal education, low wages, and low awareness of breast cancer.^6^ With incidence rates of breast cancer steadily climbing in the region, it is crucial that initiatives to improve breast cancer education be intensified both among women and throughout the health care system.

These awareness programs should be tailored to cultural norms, designed to address stigma, and, most importantly, provide a clear path to the health facility for an assessment. The Cervical Cancer Prevention Program of Zambia (CCPPZ) has increasingly used community-level social structures—traditional marriage counselors, chiefs, healers, and practitioners—to increase the uptake of health messages.^7^ Similarly, breast cancer awareness messages need to embrace locally accepted good practices. Efforts must be made to increase awareness of breast cancer symptoms among women and men, enhance their understanding of the urgency of these symptoms, allay their fears of a cancer diagnosis, and increase their ability to access care.

Building on its cervical cancer prevention platform, the Health Promotion Unit of the Zambian Ministry of Health is presently conducting breast cancer outreach programs, focusing on general health promotion messages and the early signs of the disease. However, its present outreach is too limited to achieve the goals set in the NCCSP.

### Breast Cancer Services Are Fragmented and Uncoordinated, Making It Difficult for Patients to Move Efficiently Through the Continuum of Care

The Zambian health care system has significantly improved in recent years, but gaps in cancer care remain. Services are fragmented, uncoordinated, and centralized in the capital city of Lusaka, making them difficult for patients to navigate and access. Moving from a primary care facility to a specialized cancer center can be confusing or difficult if a patient does not have proper insurance coverage, does not have enough financial resources to pay for out-of-pocket expenses, or lives a far distance from Lusaka. Some of the other barriers to care include language, cultural beliefs, stigma, and fear. Delays in diagnosis and treatment, which often stem from these barriers, ultimately lead to poorer breast cancer outcomes.

During the workshop, the discussion focused on patient navigation as a feasible instrument to reduce the time between symptom onset/recognition and start of treatment, and to help patients adhere to treatment and follow-up schemes. Patient navigation has been shown to improve breast cancer outcomes and quality of life in different global settings.^8,9^

The contribution of the civil society is key to developing sustainable public breast cancer programs. An example is the navigation program at CDH implemented by the Zambian Cancer Society and housed at CDH. Initiated in March 2017 in formal collaboration with the Ministry of Health and coordinated by the hospital social welfare unit, the navigation desk provides the following services for patients: counseling, information, appointment management, and help with follow-up and outreach. It is intended to serve patients with cancer in general, but has a strong focus on patients with cervical cancer and caregivers.

### Inadequate Human Resources Trained in Disciplines Related to Breast Cancer Control at All Levels of the Health Care System

Training of health care professionals at all levels was identified as a key issue when tackling the problem of late-stage disease presentation. It was noted that among the many issues that undergird the problem of late-stage presentation, some are women-level factors but others are related to the low level of breast cancer awareness among health care professionals who fail to appropriately refer patients for evaluation in a timely manner.

Workshop participants considered training as a key component of the solution for each challenge. Strategies to train health care providers (HCPs) at all levels and disciplines, and how to deliver training updates, were discussed in depth during the workshop. Recommendations in this area included training on patient navigation techniques, clinical breast examination (CBE), and breast cancer care (diagnosis and treatment). The tactics discussed included using the train-the-trainer model to gradually increase the number of skilled health care professionals using a standardized curriculum and appropriate training materials.

Leveraging the successes and experiences of the CCPPZ, a multidisciplinary group of Zambian health care professionals designed a clinical platform that provides multiple breast care services within a single visit.^3^ The service platform was implemented in rural areas using a breast outreach camp format and offered breast self-awareness, psychosocial counseling, CBE, breast ultrasound, ultrasound-guided biopsy, imprint cytology of biopsy specimens, and surgical treatment or referral. Overall, this initiative has proven that increased breast care capacity in Zambia is possible—even in rural settings—and if scaled, could serve to improve and shorten the delay of diagnosis and management of early-stage breast cancer at a national level.

### Breast Care Is Not Well Integrated With Other Health Services and Is Centralized in the Capital

To positively affect mortality rates, early diagnosis must be linked to effective treatment. Thus, it is paramount to develop a strategy to decentralize and coordinate—with services combined in a single facility, preferably the district hospital—comprehensive breast cancer care models that can later be scaled up.^10^

A number of investments have already been made in cancer care in Zambia. Perhaps the most obvious model for expanding and strengthening breast care is the CCPPZ, a public sector screen-and-treat approach that links screening using visual inspection with acetic acid to immediate treatment of precancerous lesions^11^ and referral of patients with suspected cervical cancer.

A key component of the program’s success has been the compression of the cervical cancer prevention pathway into a single visit, an approach that, when applied to breast care, showed improvement in time to diagnosis. The single-visit algorithms for breast and cervical cancer could thus be integrated.^12^

The central components of a new breast cancer care model evolved during the workshop and included updated needs assessment of existing health facilities at the provincial level; short- and long-term, in-country training strategies; appropriate infrastructure and equipment; procurement of essential drugs for breast cancer management; laboratory equipment and consumables, including the capacity for immunohistochemistry; establishment of a data management system; increased capacity of breast oncologists to conduct research; and rigorous periodic monitoring and evaluation of the care model. A summary of the challenges is listed in Table 1.

**TABLE 1:**
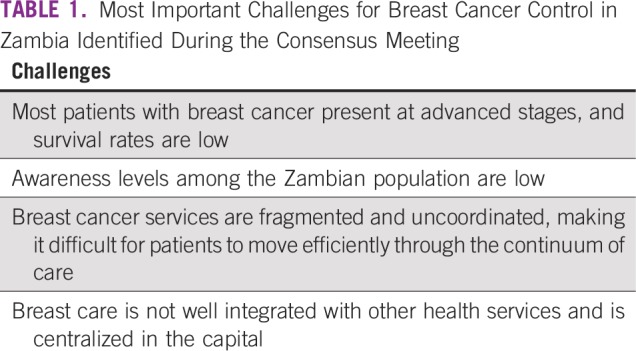
Most Important Challenges for Breast Cancer Control in Zambia Identified During the Consensus Meeting

## RECOMMENDATIONS

There was consensus that efforts should focus on the early diagnosis of breast cancer to reduce late-stage presentations as a first step toward lowering breast cancer mortality (Table 2). Initially, priority should be given to the early diagnosis of breast cancer among women presenting with clinical symptoms through annual physical breast examinations provided by HCPs. Focusing on the early diagnosis of breast cancer is an effective and affordable strategy to downstage disease. It can complement screening strategies where available and feasible.^10^

**TABLE 2:**
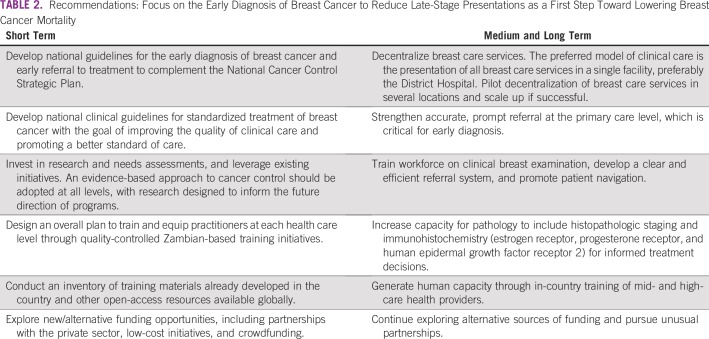
Recommendations: Focus on the Early Diagnosis of Breast Cancer to Reduce Late-Stage Presentations as a First Step Toward Lowering Breast Cancer Mortality

Because successful breast cancer control requires both early and accurate diagnosis, along with timely, accessible, and effective treatments, there was agreement that all necessary elements needed to be integrated under a program for the early diagnosis of breast cancer, led by the Ministry of Health. The program should be integrated as much as possible with the cervical cancer control program.

### Short-Term Recommendations

- Develop national guidelines for the early diagnosis of breast cancer and early referral to treatment. This should be the document used to complement the NCCSP and to provide guidance on the implementation of a cost-effective national breast cancer early-diagnosis program. On the basis of breast cancer control recommendations from WHO, these guidelines should provide a framework for how to implement simple and affordable strategies, adapted and tailored to individual programs or health facilities, within the parameters of what is acceptable in Zambia. The guidelines should provide the framework for making quality services available in the country, guiding providers at all levels within the health care system in effective, evidence-based interventions that are integrated with service platforms for other cancers affecting women.
- Develop national clinical guidelines for standardized treatment of breast cancer with the goal of improving the quality of clinical care and promoting a better standard of care, providing guidelines that help ensure consistent treatment and reduce unnecessary variation in care. They could be based on existing standards and adapted to the context of Zambia’s resources, cultural beliefs, and health care practices. Evidence from studies conducted in Zambia and other African countries should receive higher valuation for the formulation of Zambian national guidelines.
- Invest in research, needs assessments, and approaches that leverage existing initiatives. All new interventions should be evidence based and contextually appropriate on the basis of current needs and existing assets. Research should be used to inform the future directions of programs.

Recommendations in this area include a descriptive analysis of breast cancer epidemiology and patterns of care in Zambia, improvement of the cancer registry and electronic recording mechanisms, and inventory of existing training opportunities in the country and online. Because funding may not be readily available, we suggest revising existing assessments and using low-cost or free resources, such as the Breast Cancer Initiative 2.5 assessment tools (https://www.fredhutch.org/en/labs/phs/projects/breast-cancer-initiative_2-5/assessment-tools.html).

- Explore new/alternative funding opportunities, including partnerships with the private-sector, low-cost initiatives, and crowdfunding.
- Design an overall plan to train and equip practitioners at each health care level through quality-controlled Zambian-based training initiatives. Training should be considered a key part of the solution for breast cancer control. Recommendations in this area include training for health promoters and patient navigators, CBE for HCPs, and specialized breast care for others (diagnosis and treatment). For cost savings, examine centralizing training across all interventions.
- Conduct an inventory of training materials already developed in the country and other open-access resources available. Some trainings will overlap across types of health care practitioners, and others will be able to integrate with existing trainings, for example, cervical cancer, HIV, or reproductive health.

### Medium- and Long-Term Recommendations

- Decentralize breast care services. The preferred model of clinical care is the presentation of all breast care services in a single facility, preferably the district hospital. Pilot decentralization of breast care services in several locations and scale up if successful. Implement initiatives that incrementally build capacity to deliver prompt diagnostic, treatment, and supportive care services integrated with existing services targeting equity in women’s health.
- Strengthen accurate, prompt referral at the primary care level, which is critical for early diagnosis. Train the workforce on CBE, develop a clear and efficient referral system, and promote patient navigation.
- Increase capacity for pathology to include histopathologic staging and immunohistochemistry (estrogen receptor, progesterone receptor, and human epidermal growth factor receptor 2) for informed treatment decisions. Accurate clinical and pathologic work-up of a biopsy sample is required for a definitive diagnosis. The timely reporting of breast diagnostic tests to the appropriate provider and patient is critical to improving outcomes. It is important to formally assess existing pathology and laboratory capacity in both the private and public sectors and explore the option of contracting private-sector pathology services to increase pathology capacity overall. Investigate the possibility of implementing telepathology and other teleoncology service platforms in collaboration with global partners (teleradiology, telesurgery, and so on).
- Generate human capacity through in-country training of mid- and high-care health providers. Take advantage of existing opportunities offered by international organizations or academic institutions for short-term training, such as fellowships, online seminars, and so forth. Customize trainings by assessing the educational needs and assets of communities before developing educational interventions, using established tools such as focus groups, surveys, interviews, and needs assessments.
- Continue exploring alternative sources of funding and pursue innovative partnerships. When designing trainings or interventions, borrow from other cancers, diseases, or challenges. For example, for the success of scaling cervical cancer prevention services in Africa, it has been critical to streamline services (One Stop Shop), by linking screening with opportunities for immediate treatment or referral, that is, screen and treat or refer.

## NEXT STEPS

The recommendations and initiatives that emerged from the consensus meeting align with a resource-stratified, phased-implementation approach to breast cancer detection, diagnosis, and treatment that has been proposed for similar settings.^13^

As a first step, the breast health care system must have the capacity to manage clinical detectable breast cancers coupled with a strong community education and mobilization effort before any screening program—mammographic or clinical—is implemented. Only a system with the capacity and infrastructure to manage clinical/palpable disease will be able to manage the increase in numbers of asymptomatic patients (International guidelines from WHO recommend delaying the introduction of population-based mammography screening in the public health system until processes are in place to effectively detect, diagnose, and treat palpable tumors).

Health care leaders in Zambia could begin by leveraging results from the co-creation workshop to gain political support and advocate for a strong policy framework in support of a context-appropriate breast care model that adequately addresses current needs and builds on existing assets. Protocols and guidelines should be standardized for the early diagnosis and treatment of breast cancer, and health care professionals should be educated according to the guidelines. This will allow for an efficient and systematic triage and diagnosis of clinical breast disease, and stage-appropriate treatment planning, adapted to the available resources.

Efforts are under way through multiple stakeholders to implement recommendations related to the development of national guidelines for the early diagnosis of breast cancer, training of breast surgeons, and epidemiologic research, including the improvement of electronic recording mechanisms.

Civil society, working collaboratively with the government and the private sector, can support these efforts by shaping programs that are responsive to the needs, raise awareness, and influence decision makers to bring necessary transformative change. Country ownership is absolutely needed to sustain the program in the long term, along with political stewardship and accountability.

Finally, strong and effective international partnerships are needed to bring together complementary resources to address the breast cancer burden. Zambian stakeholders may seek these alliances in the early stages of the development of the program and during the planning process to secure varied sources of funding to meet national priorities.
